# An External Selection Mechanism for Differential Evolution Algorithm

**DOI:** 10.1155/2022/4544818

**Published:** 2022-04-04

**Authors:** Haigang Zhang, Da Wang

**Affiliations:** School of Software, Yunnan University, Kunming 650000, China

## Abstract

The procedures of differential evolution algorithm can be summarized as population initialization, mutation, crossover, and selection. However, successful solutions generated by each iteration have not been fully utilized to our best knowledge. In this study, an external selection mechanism (ESM) is presented to improve differential evolution (DE) algorithm performance. The proposed method stores successful solutions of each iteration into an archive. When the individual is in a state of stagnation, the parents for mutation operation are selected from the archive to restore the algorithm's search. Most significant of all, a crowding entropy diversity measurement in fitness landscape is proposed, cooperated with fitness rank, to preserve the diversity and superiority of the archive. The ESM can be integrated into existing algorithms to improve the algorithm's ability to escape the situation of stagnation. CEC2017 benchmark functions are used for verification of the proposed mechanism's performance. Experimental results show that the ESM is universal, which can improve the accuracy of DE and its variant algorithms simultaneously.

## 1. Introduction

Differential evolution (DE) algorithm [1], introduced by Storn and Price in 1995, is one of the most popular evolutionary algorithms. DE is easy to be understood, and the principle is simple. Still, it demonstrates good optimization ability and is used in many single-objective optimization problems successfully, including continuous optimization [2], discrete optimization [3], constrained optimization [4], and unconstrained optimization [5].

Different from other meta-heuristic algorithms [6], the procedures of differential evolution algorithms can be summarized as population initialization, mutation, crossover, and selection. After each iteration, promising solutions with better fitness values are selected to survive to the next iteration. These solutions can be called successful solutions. DE repeats this procedure until a predefined termination criterion is reached. However, DE may suffer stagnation during the evolution process, which means stop generating successful solutions and converging to a fixed point [7]. When the population stagnates, taking appropriate strategies to restore the search should theoretically further improve the algorithm's performance.

Successful solutions are generally located in valleys of the fitness landscape. As the iteration progresses, successful solutions containing useful information may be discarded and underutilized. We, therefore, propose an external selection mechanism (ESM). First, ESM stores successful solutions of each iteration into an archive. When the individual is in a state of stagnation, the parents for mutation operation are selected from the archive. In this way, the population can regain diversity and restore searchability. Second, the diversity and superiority of the archive are relatively significant because they can directly affect the performance of the algorithm. Therefore, a rule is proposed to update the archive based on novel crowding entropy diversity measurement in the fitness landscape and fitness rank.

To verify and analyze the effectiveness of the ESM, we performed a series of experiments and comparisons on the CEC2017 benchmark set [8], incorporating three classic DEs and five state-of-the-art DE variants. Results indicate that the ESM can effectively improve the algorithm performance without increasing the computational complexity.

The rest of this study is organized as follows: Section 2 introduces the canonical DE algorithm, including its typical mutation, crossover, and selection operators.  Section 3 reviews related work. The proposed ESM procedures are introduced in Section 4. The effectiveness of the proposed mechanism is discussed in Section 5 based on the computational results. Conclusions and future work are illustrated in Section 6.

## 2. Differential Evolution

In this section, we introduce the basic differential evolution algorithm, including the well-known mutation strategy DE/rand/1 [1] and other widely used mutation strategies.

### 2.1. Initialization

An initial random population *P* consists of NP individuals, each represented by {*X*_*i*_^*t*^=(*x*_*i*,1_^*t*^, *x*_*i*,2_^*t*^,…, *x*_*i*,*D*_^*t*^)*|i*=1,2,…, NP}, where *t*=0,1,2,…, *T*_max_ is the iteration number, and *D* is the number of dimensions in the solution space. In DE, uniformly distributed random functions are used to generate initial solutions *x*_*i*,*j*_^0^=*x*_*j*,min_+rand(0,1) · (*x*_*j*,max_ − *x*_*j*,min_), where *x*_*j*,max_ and *x*_*j*,min _ are the maximum and minimum boundary values, respectively, on the corresponding *j* th dimension.

### 2.2. Mutation

At iteration *t*, for each target vector *X*_*i*_^*t*^, a mutant vector *V*_*i*_^*t*^ is generated according to the following:

(1) $$\begin{array}{c} \text{DE}/\text{rand}/1:V_{i}^{t}=X_{r_{1}}^{t}+F\cdot \left(X_{r_{2}}^{t}-X_{r_{3}}^{t}\right). \end{array}$$

Other widely used mutations strategies include

(2) $$\begin{array}{c} \frac{\text{DE}}{\left(\text{best}/1\right)}:V_{i}^{t}=X_{\text{best}}^{t}+F\cdot \left(X_{r_{1}}^{t}-X_{r_{2}}^{t}\right),\\ \frac{\text{DE}}{\left(\text{best}/2\right)}:V_{i}^{t}=X_{\text{best}}^{t}+F\cdot \left(X_{r_{1}}^{t}-X_{r_{2}}^{t}\right)+F\cdot \left(X_{r_{3}}^{t}-X_{r_{4}}^{t}\right),\\ \frac{\text{DE}}{\left(\text{rand}/2\right)}:V_{i}^{t}=X_{r_{1}}^{t}+F\cdot \left(X_{r_{2}}^{t}-X_{r_{3}}^{t}\right)+F\cdot \left(X_{r_{4}}^{t}-X_{r_{5}}^{t}\right),\\ \frac{\text{DE}}{\left(\text{current}-\text{to}-\text{best}/1\right)}:V_{i}^{t}=X_{i}^{t}+F\cdot \left(X_{\text{best}}^{t}-X_{i}^{t}\right)+F\cdot \left(X_{r_{1}}^{t}-X_{r_{2}}^{t}\right),\\ \frac{\text{DE}}{\left(\text{current}-\text{to}-\text{rand}/1\right)}:V_{i}^{t}=X_{i}^{t}+F\cdot \left(X_{r_{1}}^{t}-X_{i}^{t}\right)+F\cdot \left(X_{r_{2}}^{t}-X_{r_{3}}^{t}\right), \end{array}$$

where *r*_1_, *r*_2_, *r*_3_, *r*_4_, and *r*_5_ are mutually different integers randomly generated from the range (1,2,…, NP), and they are different from *i* (*r*_1_ ≠ *r*_2_ ≠ *r*_3_ ≠ *i*). *X*_best_^*t*^ is the individual vector with the best fitness value in the population at iteration *t*.

### 2.3. Crossover

We illustrate the binomial crossover, in which the target vector *X*_*i*_^*t*^ and donor vector *V*_*i*_^*t*^ exchange elements according to the following rules:

(3) $$\begin{array}{c} u_{i,j}^{t}=\left\{\begin{array}{cc} v_{i,j}^{t},\;\text{if }\left(\text{rand}\left(0,1\right)<\text{CR or }j=j_{\text{rand}}\right),\\ x_{i,j}^{t}, & \text{otherwise,} \end{array}\right. \end{array}$$

where the crossover rate CR ∈ [0,1] is a uniformly distributed random integer in [1, *D*] that ensures at least one element of the trial vector is inherited from the donor vector.

### 2.4. Selection

The greedy selection strategy is generally used in DE. The variable, *X*_*i*_^*t*+1^, is assigned when the fitness value of the trial vector, *U*_*i*_^*t*^, is equal to or better than *X*_*i*_^*t*^, and otherwise *X*_*i*_^*t*^ is reserved:

(4) $$\begin{array}{c} X_{i}^{t+1}=\left\{\begin{array}{cc} U_{i}^{t}, & f\left(U_{i}^{t}\right)\leq f\left(X_{i}^{t}\right),\\ X_{i}^{t}, & \text{otherwise,} \end{array}\right. \end{array}$$

where *f*(*x*) is the fitness function.

## 3. Related Work

Most research on improving DE has focused on mutation operator [9]. In recent decades, there have been many mutation operators with distinct search performance that have been proposed. Fan et al. [10] proposed a triangular mutation strategy, which was considered a local search operator. Zhang et al. [11] introduced a new DE variant, JADE, improving optimization performance by a new mutation strategy, DE/current − to − *p*best/1. DE/current − to − *p*best/1 is one of the most successful mutation operators because of its relatively balanced performance between exploration and exploitation. Wang et al. [12] proposed a new mutation strategy called DE/current − to − *l*best/1 based on the values near the current parameter to keep balance of exploitation and exploration capabilities during the differential evolution. A novel DE algorithm with intersect mutation operation called intersect mutation differential evolution (IMDE) was proposed [13] to further improve the performance of the standard DE algorithm. Mohamed et al. [14] proposed two mutation strategies, DE/current − to − ord_best/1 and DE/current − to − ord_*p*best/1. Both of the proposed mutation strategies were based on ordering three selected vectors to achieve different search behaviors. Deng et al. [15] proposed a novel DE variant called DCDE based on a dynamic combination-based mutation operator to achieve an appropriate balance between global exploration ability and local exploitation ability.

In addition, novel neighborhood-based [16], dimension-based [17], opposition-based [18], and regeneration-based [19] mechanisms also showed their effectiveness on improving the performance of DE and its variants. In this study, we focus on the related work on archive-based techniques for DE. In the multiobjective evolutionary algorithm, a subpopulation called the external archive is used to store nondominated solutions that have been found during the search [20, 21], employing an archive is almost standard in multiobjective optimization [22]. The goal of single-objective global numerical optimization is to find a global optimum in decision space of a given objective function. Recently, archive is also introduced into global numerical optimization. In JADE [11], a set of recently explored inferior solutions are archived, and their difference from the current population is considered as a promising direction toward the optimum. The archive is applied to the second difference vector of the mutation strategy in JADE. Zhou et al. [23] proposed a DE framework with guiding archive (GAR-DE), GAR-DE chose the base vector of mutation strategy from the archive to help DE escape from the situation of stagnation, and Manhattan distance metric was used to maintain the diversity of the archive. Zeng et al. [24] proposed a new selection operator (NSO) for DE, which archived not only the successful updated solutions but also the discarded trial solutions. The NSO focused on selecting appropriate solution from the archive to survive to the next generation.

As can be seen from preceding explanation, the differences among archive-based mechanisms mainly consist in two aspects: what information/solutions should be stored in the archive; how to maintain and update the archive; and how to use the archive to guide the search. The most striking feature of the proposed ESM is the use of information entropy rather than distance metric [23, 25] to maintain the diversity of the archive.

Shannon defined the information entropy theory [26] for the first time in 1948. In information theory, entropy is used to measure the expected value of a random variable. As the evaluation scale of information quantity of stochastic process, information entropy has been extended to a general and effective tool to solve difficult numerical problems and uncertain polynomial combinatorial optimization problems [27, 28]. An information theoretic technique was adopted to analyze the ruggedness of a continuous fitness landscape in [29, 30]. Petalas et al. [27] proposed a memetic particle swarm optimization algorithm that exploits Shannon's information entropy for decision-making in swarm level, as well as a probabilistic decision-making scheme in particle level, for determining when and where local search is applied. As can be seen from the previous studies, entropy actually reflects the degree of chaos of system. Therefore, entropy principle can be a promising method applied to evolutionary algorithm to ensure population diversity.

## 4. The Proposed ESM

In this section, we discuss the characteristics of the successful solution archive and archive-updating procedure and describe the ESM implementation.

### 4.1. Successful Solution Archive

**Figure 1:**
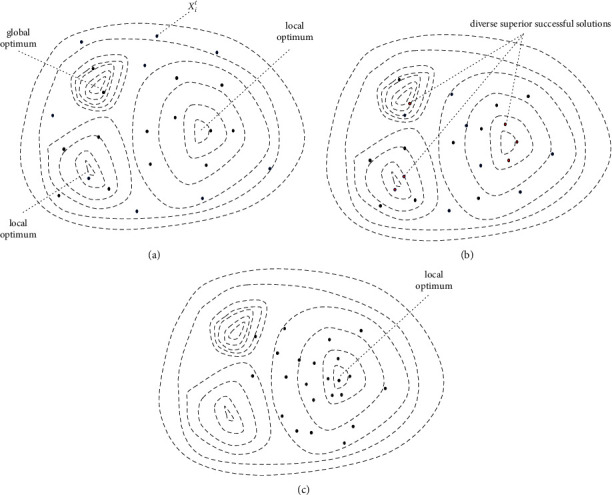
Illustration of the successful solution distribution in a two-dimensional space: (a) at the initial stage; (b) at the intermediate stage; (c) at the terminal stage.

From Section 2, we can know that DE selects one vector from the trial vector and parent vector to survive to the next generation, and the survivor is known as the successful solution. As shown in Figure 1(a), in the two-dimensional solution space of a multimodal problem, the population is evenly distributed at the initial stage. As the iteration continues, from Figure 1(b) we can conclude that superior successful solutions (red points) can be generated in possible optimal regions. However, in Figure 1(c), the random and greedy selection operation makes the population gradually converge to a local optimum. Some successful solutions may be discarded without being fully exploited because of the greedy selection operation as the iteration progresses. An appropriate mechanism to utilize the successful solutions with high diversity contribution can make the algorithm reexplore the missed potential optimal solution. To some extent, it is also a remedy for the greedy selection operation.

**Figure 2:**
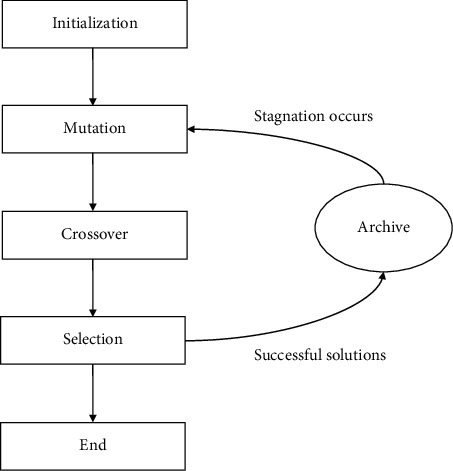
Illustration of the successful solution archive.

Considering the above analysis, as depicted in Figure 2, an external archive is designed to store successful solutions generated during each iteration. When an individual is in a state of stagnation, the archive is activated for parent selection of mutation operation. For instance, the classic mutation strategy DE/rand/1 can be modified to

(5) $$\begin{array}{c} V_{i}^{t}=X_{ar_{1}}^{t}+F\cdot \left.\left(X_{ar_{2}}^{t}-X_{ar_{3}}^{t}\right)\right.,i\varepsilon I, \end{array}$$

where *ar*_1_, *ar*_2_, *ar*_3_ ∈ *A*^*t*^∧*ar*_1_ ≠ *ar*_2_ ≠ *ar*_3_, *A*^*t*^ is the successful solution archive at iteration *t*. A simple but efficient method is adopted at this point, when the number of steps for the individual's fitness value to stop updating reaches a predetermined value *Q*, the individual is considered in a stagnant state [31].

### 4.2. Archive Updating Procedure

As the successful solution archive has been established, the current problem is how to update the archive to keep its diversity and superiority in the whole evolution process. An archive containing diverse successful solutions can drag the individual out of stagnation without crippling the algorithm's performance.

#### 4.2.1. Individual Diversity Measure

To our best knowledge, in single-objective optimization, diversity is usually defined by the spatial distribution of the whole population [32], but there is few diversity contribution measures for a single individual to whole population. Wang et al. [33] proposed an Euclidean distance-based diversity metric for each individual, but a huge computational cost is needed to calculate the distance between every two individuals. To utilize diversity information while reducing the computational cost, we can estimate the diversity by fitness value distribution on individual level. Fitness distance can represent the spatial distance to some extent [34].

**Figure 3:**
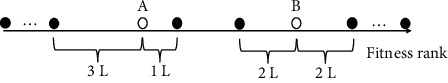
Crowding distance presented in fitness landscape.

In multiobjective optimization, Deb et al. [35] proposed a crowding distance measure method to get an estimation of the density of solutions surrounding a particular solution in the population, by calculating the average distance of two points on either side of this point along each of the objectives. However, the distribution of this point is not considered, and the only average distance may not accurately reflect the crowding degree. With utilizing the characteristic of fitness distance, we can extend the crowding distance measure to single-objective optimization. For example, in Figure 3, the crowding distance of the solution A can be calculated as 3*L*+1*L*=4*L* (according to crowding distance calculation method in [35]), which is the same as the solution B (2*L*+2*L*=4*L*), whereas solution B obviously has a higher diversity contribution than solution A.

#### 4.2.2. Archive Update Procedure

**Figure 4:**
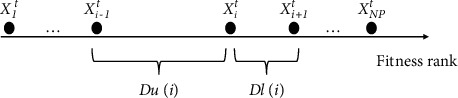
Crowding entropy presented in fitness landscape.

From the above statement, this study proposes a crowding entropy diversity measurement in the fitness landscape at individual level. As shown in Figure 4, we sort the archive population in the ascending order of fitness during each iteration, then, a fitness distance estimation operator can be formulated as follows:

(6) $$\begin{array}{c} Dl\left(i\right)=\left|f_{i+1}-f_{i}\right|, \end{array}$$

(7) $$\begin{array}{c} Du\left(i\right)=\left|f_{i}-f_{i-1}\right|, \end{array}$$

where *f*_*i*_ is the fitness value of the *i* th individual. *Dl*(*i*) and *Du*(*i*) can be further normalized, then, the crowding entropy of the *i* th individual can be defined as follows:

(8) $$\begin{array}{c} P_{i1}=\frac{Dl\left(i\right)}{Dl\left(i\right)+Du\left(i\right)}, \end{array}$$

(9) $$\begin{array}{c} P_{i2}=\frac{Du\left(i\right)}{Dl\left(i\right)+Du\left(i\right)}, \end{array}$$

(10) $$\begin{array}{c} {\text{CE}}_{i}=-\sum_{j=1}^{2}P_{ij}\log_{2}\left(P_{ij}\right), \end{array}$$

where CE_*i*_ ∈ [0,1], the larger CE means that the individual distribution is more uniform, so it has a higher diversity contribution. According to Figure 3, we can further calculate CE_*A*_=0.8 and CE_*B*_=1, this is also consistent with their diversity contribution. Since the best and worst individuals locate in the boundary scope, there is only one neighbor around them, respectively. Therefore, we set CE_1_=CE_2_ and CE_NP_=CE_NP−1_ for the ESM to function normally. To keep the superiority of the archive, fitness value rank is further introduced:

(11) $$\begin{array}{c} {\text{FR}}_{i}=i,\;i=1,2,\ldots ,\text{NP}, \end{array}$$

(12) $$\begin{array}{c} R_{i}=\left(1-\frac{{\text{FR}}_{i}}{\text{NP}+1}\right)\cdot {\text{CE}}_{i}, \end{array}$$

where *R*_*i*_ represents the priority of the individual to be updated. An individual in the archive with a higher rank (i.e., far from the global optimum) or with lower entropy (i.e., located in a dense area) should have a higher probability of being updated.

### 4.3. The Implementation of the ESM

Based on the ESM described above, we present the ESM updating procedure in Algorithm 1 and the complete implementation based on classic DE with mutation strategy DE/rand/1 in Algorithm 2. Different from the existing alternative for the selection of parents [33, 36], the proposed mechanism is an adaptive approach that considers the feedback of the recently successful solutions so that the robustness of the DE algorithm has the potential to be enhanced by dynamically adapting promising parents for the situation of stagnation. Moreover, the proposed ESM is computationally efficient. The time complexities of updating operations of the archive are *O*(NP). Additionally, the selection of parents, counting of the consecutive unsuccessful updates, and stagnation detection do not increase the overall time complexity.

## 5. Experiments and Comparisons

In this section, the proposed ESM is first integrated into three representative classic DE algorithms, including DE/rand/1, DE/best/1, and DE/current − to − best/1, to verify its efficiency. Then, five state-of-the-art DE variants, including j2020 [37], EJADE [38], BeSD [39], EBSHADE [14], and EBLSHADE [14], are introduced to incorporate with the ESM for further comparison. Finally, all experiments are conducted on the CEC2017 benchmark set [8], which contains 30 representative benchmark functions in four categories: unimodal (F1–F3), simple multimodal (F4–F10), hybrid (F11–F20), and composition (F21–F30). This benchmark set is widely used in the performance testing of evolutionary algorithms [9]. It is worth mentioning that the ESM does not change existing mechanisms in algorithms including the bound constraint handling and linear population size reduction.

The parameters of all algorithms are listed in Table 1. The common parameters of the incorporated algorithms are set as follows: The population size NP is 100, the stagnation tolerance *Q* is 120, and the maximum allowed number of function evaluations Max_FE*s*=10000*∗D*, where *D* is the problem dimension. Moreover, *H* represents the historical memory size associated with the adaptive control of scaling factors (*F*) and crossover rates (CR), LP is the learning period.

Limited by the article space, this study presents results at *D*=30. For the fairness of comparison, each algorithm was implemented in MATLAB 2017b and executed over 51 independent runs on a Windows 10 64 bit desktop PC with 32 GB of RAM and a 3.0 GHz Intel Core i7-9700 processor.

### 5.1. Improving the Performance of Classic DEs and State-of-the-Art DE Variants

The following tables show the mean value (Mean) and standard deviation (Std.Dev) of the error value *f*(*X*) − *f*(*X*^*∗*^), where *f*(*X*^*∗*^) representing the global optimum. The “+,” “−,” and “=” signs at each row of the table indicates that the ESM-based algorithm is, respectively, better than, equal to, or worse than the comparison algorithm.

Tables 2–4 show the performance of three classic DEs. For DE/rand/1, which is widely recognized for its diversity maintaining capability and relative low search efficiency, the ESM significantly improves its performance on 24 test functions and only cause performance degradation on two test functions, as shown in Table 2. DE/best/1 is well known as its local exploitation capability and fast convergence speed, the ESM also makes progress on 19 functions, as presented in Table 3. Relatively, the performance of ESM-DE/best/1 decreases on five functions including F13, F16, F24, F27, and F29 and keeps the same as DE/best/1 on the rest six functions. DE/current − to − best/1 is more inclined to balance global exploration and local exploitation. From Table 3, we can find that the ESM also achieves improvement on 26 functions. The results of ESM-DE/current − to − best/1 only decrease on two functions including F2 and F19. With functions F25 and F27, the two competitors keep the same.

From the above experiments, we can conclude that the ESM can significantly improve classic DE's performance. To further verify the ESM's universality, five representative and advanced DE variants are introduced, Tables 5–9 show these variants and their competitors' performance. For ESM-j2020, as shown in Table 5, significant improvements are achieved on 23 functions. From Tables 6to 9, we know that the number of functions with “+” decreases compared with the results shown in Tables 2–5. Meanwhile, from Tables 6to 9, only a few functions show performance degradation after applying the ESM. We can therefore conclude that the ESM is still competitive even in recognized excellent DE variants.

We further conducted Wilcoxon signed-ranked test with a 0.05 significance [40] to statistically verify the effectiveness of the ESM. In Table 10, the *p*-value obtained by ESM-DE/rand/1, ESM-DE/best/1, ESM-DE/current − to − best/1, ESM-j2020, and ESM-BeSD are all less than 0.05, which indicates a significant improvement is achieved by the proposed ESM. It also can be found that ESM-EJADE, ESM-EBSHADE, and ESM-EBLSHADE still maintain their advantages in terms of the value of *R*+ and R–, which also reflects that the ESM achieves more promising results.

As mentioned before, the functions of CEC2017 can be divided into four categories: unimodal functions, simple multimodal functions, hybrid functions, and composition functions. To analyze whether the performance of the ESM is related to the type of function, the performance of the ESM-based algorithms for each type of function category are presented in Table 11. As shown in Table 11, the ESM performs best on hybrid functions. Following the adoption of the ESM, the algorithm has an 81.25% probability of finding a better solution but only a 16.25% probability of finding a worse solution on hybrid functions. Besides, the performance of the ESM is almost the same on simple multimodal functions and composition functions and relatively worst on unimodal functions. Table 11 demonstrates that if the optimization problem is multimodal function, hybrid function, or composition function, then the proposed selection mechanism can be considered in the DE algorithm.

### 5.2. Convergence Analysis

**Figure 5:**
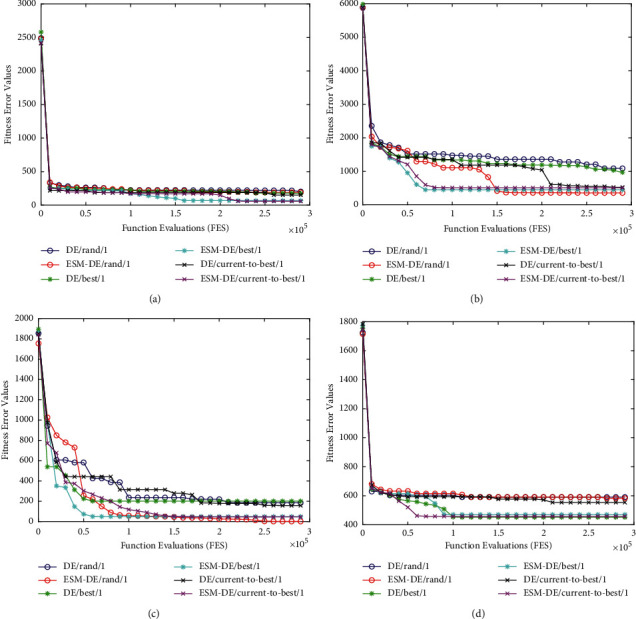
Convergence curves of three classic DEs and relative ESM-DEs on four 30-D CEC2017 benchmark functions: (a) F7; (b) F16; (c) F20; (d) F24.

To more clearly show the influence of the ESM on algorithm convergence and avoid diversion, four representative functions from CEC2017 are extracted, including F7, F16, F20, and F24, convergence curves for these functions with the three classic DEs and their relative ESM-based versions are presented. F7 is a simple multimodal function with a characteristic of convexity. From Figure 5(a), we can find that all six comparison algorithms have a similar convergence rate; this also reflects that the ESM does not cripple the convergence speed of the algorithm on F7. From Figures 5(b)to 5(d), it can be seen that when each algorithm adopts the proposed ESM, it converges to a better value than the original algorithm with faster convergence. This is mainly because the algorithm can hardly escape when it suffers stagnation. The algorithm that uses the proposed ESM has a more excellent capability to keep search efficiency.

### 5.3. Population Diversity Analysis

As the ESM adopts the entropy-based individual diversity measure, we further calculated the diversity of the main population and archive population on F7, F16, F20, and F24 for three classic DEs. It should be pointed out that we used the concept proposed in [41] to measure population diversity:

(13) $$\begin{array}{c} \bar{x_{j}}=\frac{1}{\text{NP}}\sum_{i=1}^{\text{NP}}x_{j,i},\\ \text{Population Diversity}=\sqrt{\frac{1}{\text{NP}}\overset{\text{NP}}{\underset{i=1}{\sum }}\overset{D}{\underset{j=1}{\sum }}{\left(x_{j,i}-\bar{x_{j}}\right)}^{2}}. \end{array}$$

**Figure 6:**
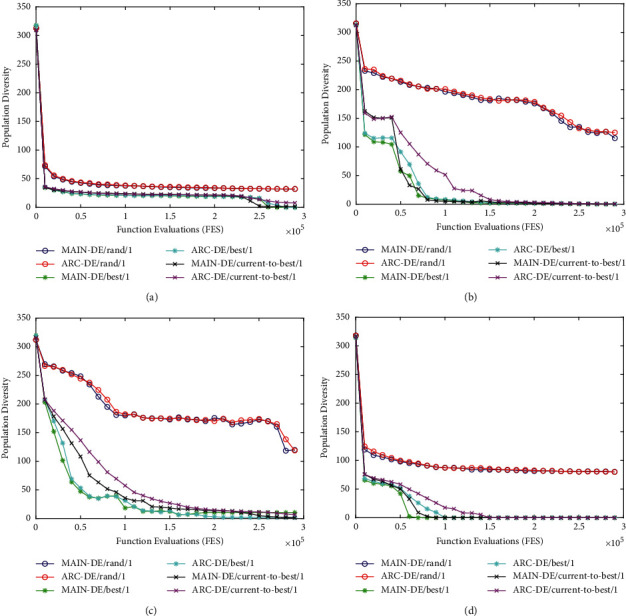
Population diversity curves of main population and archive population of three classic DEs on four 30-D CEC2017 benchmark functions: (a) F7; (b) F16; (c) F20; (d) F24.

As illustrated in Figure 6(a), the main population and archive population have an almost uniform diversity on F7. From Figures 6(b)to6(d), it can be concluded that the archive population can maintain a relatively higher diversity than the main population at the intermediate stage for DE/current − to − best/1 and DE/best/1. To sum up, the archive population can maintain relative diversity during the search process, which makes it possible for the algorithm to recover from stagnation.

### 5.4. Parameter Sensitivity Analysis

**Figure 7:**
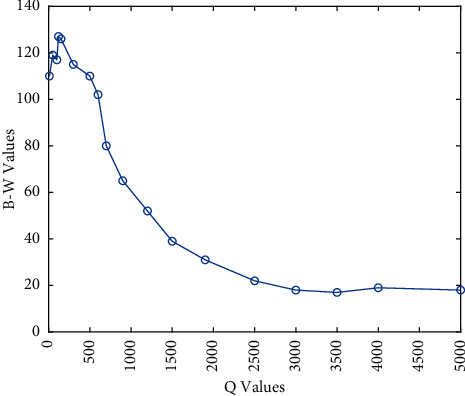
The B-W values at different Q values.

As illustrated in the previous sections, the ESM contains one parameter *Q* required adjustment. *Q* is a threshold which represents the stagnation steps.  Table 12 shows the B-W values that represent the variation between better (“+”) and worse (“−”) number of the mean value after applying the ESM. It can be seen that the ESM-based algorithm performance is not so sensitive to the change of *Q* values over a finite range. For further comparison, A wider range of *Q* values and its corresponding B-W values are shown in Figure 7. In general, when the *Q* value is too large, the overall performance of the ESM begins to deteriorate. This is mainly because too large *Q* value reduces the probability of the ESM intervening in the algorithm; therefore, it is difficult for the ESM to function in limited iterations. In summary, considering the overall performance of the ESM, *Q* is set to 120.

### 5.5. Scalability Analysis

**Figure 8:**
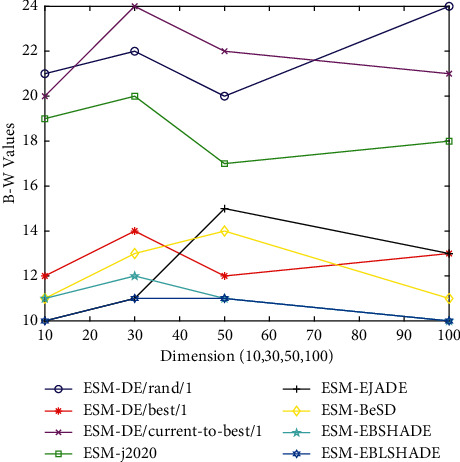
The curves of B-W values for the ESM-based algorithm compared to the original algorithm.

The dimension of the test functions governs the difficulties on finding the global optimum. Higher dimensional functions are generally more difficult to solve. To verify the relationship between the dimensionality and the performance of the proposed mechanism, we evaluate the average performance of eight ESM-based DE algorithms in CEC2017 benchmark set.  Table 13 shows the results (+/=/−) of the considered algorithms at *D*=10, 30, 50, and 100 on the test functions, for intuition, we convert the corresponding B-W values into Figure 8. We can find that the performance of the ESM-based algorithm fluctuates slightly but does not degrade significantly with the increase of problem dimension compared to the original algorithm. In Table 14, the Friedman test [40], a widely used nonparametric test in the EA community, is used to validate the performance of all algorithms based on the mean value. It is not difficult to see that the *p*-values from *D*=10 to *D*=100 calculated by the Friedman test are all less than 0.05. Therefore, there are significant differences in the performance of the comparison algorithms on the corresponding dimensions, and ESM-EBLSHADE gets the first rank overall.

## 6. Conclusion and Future Work

In DE, making full use of the successful solutions generated in iterations is a meaningful work to improve algorithm performance. In this study, an external selection mechanism (ESM) is proposed to restore the searchability of the algorithm when an individual is in a state of stagnation. The ESM mainly contains a successful solution archive mechanism and a crowding entropy diversity control strategy. It can be easily integrated into the existing DE algorithms to improve its performance further.

Experiments are conducted on the CEC2017 benchmark sets cooperated with three classic DEs and five state-of-the-art DE variants. From experiment results, we can see that the ESM-based algorithm can significantly improve the original algorithm's solution accuracy; the ESM also does not increase the computational complexity of the original algorithm due to the introduction of entropy. Further, experiments also show that ESM has a fairly positive effect on multimodal function, hybrid function, and composition function, its scalability on different problem dimensions also has a certain universal.

Notably, experimental results proved that the ESM can effectively improve various DE variants' performance, more study is required. Therefore, our future work will mainly focus on (1) improving the ESM to make it suitable for various single-objective DE; (2) researching the application of the proposed ESM in other evolutionary algorithms.

## Figures and Tables

**Algorithm 1:**
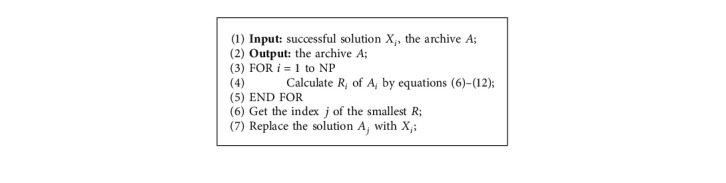
The pseudo code of ESM updating procedure.

**Algorithm 2:**
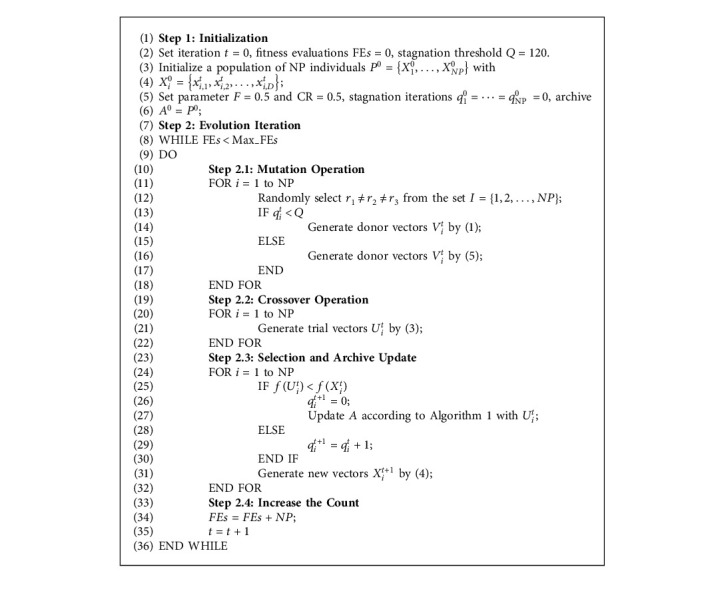
The pseudo code of DE/rand/1 with the proposed ESM.

**Table 1:** Parameter settings of all compared algorithms.

No.	ESM	*A*=NP, *Q*=120
1	DE/rand/1	*F*=0.5, CR=0.9;
2	DE/best/1	*F*=0.5, CR=0.5;
3	DE/current − to − best/1	*F*=0.5, CR=0.5;
4	j2020 [37]	*F*=0.5, CR=0.9;
5	EJADE [38]	*u*_*F*_=0.5, *u*_CR_=0.5, *c*=0.1, *p*=0.05;
6	BeSD [39]	*K*=0.5;
7	EBSHADE [14]	*M*_*F*_=0.5, *M*_CR_=0.5, and *H*=100;
8	EBLSHADE [14]	*M*_*F*_=0.5, *M*_CR_=0.5, and *H*=100;

**Table 2:** Comparison results of DE/rand/1 on CEC2017 test suite (*D*=30).

	DE/rand/1		ESM-DE/rand/1		
	M*E*an	Std.D*E*v	M*E*an	Std.D*E*v	
F1	5.8903*E* − 01	2.9710*E* − 01	**4.5086E − 01**	1.7552*E* − 01	+
F2	5.7444*E* + 23	9.9418*E* + 23	**9.2675E + 21**	2.5025*E* + 22	+
F3	3.4902*E* + 04	6.1469*E* + 03	**2.3752E + 04**	3.9591*E* + 03	+
F4	**6.5749E + 01**	9.5494*E* + 00	6.7267*E* + 01	1.0601*E* + 01	−
F5	1.7859*E* + 02	9.8332*E* + 00	**1.7115E + 02**	1.1998*E* + 01	+
F6	1.3560*E* − 06	1.8981*E* − 06	6.2629*E* − 07	4.5960*E* − 07	=
F7	2.1873*E* + 02	1.0943*E* + 01	**2.1104E + 02**	8.6729*E* + 00	+
F8	1.8310*E* + 02	9.0762*E* + 00	**1.7903E + 02**	7.5831*E* + 00	+
F9	0.0000*E* + 00	0.0000*E* + 00	0.0000*E* + 00	0.0000*E* + 00	=
F10	6.8115*E* + 03	2.7719*E* + 02	**6.7908E + 03**	2.7506*E* + 02	+
F11	7.7115*E* + 01	1.8139*E* + 01	**7.6014E + 01**	2.7738*E* + 01	+
F12	1.9220*E* + 06	1.3261*E* + 06	**1.3328E + 05**	1.6493*E* + 05	+
F13	6.9480*E* + 02	1.3966*E* + 02	**4.9388E + 02**	8.5027*E* + 01	+
F14	7.6534*E* + 01	6.7506*E* + 00	**7.3477E + 01**	6.1189*E* + 00	+
F15	7.2091*E* + 01	6.3029*E* + 00	**6.0288E + 01**	7.8284*E* + 00	+
F16	1.1650*E* + 03	1.6343*E* + 02	**9.3936E + 02**	1.6736*E* + 02	+
F17	2.8488*E* + 02	6.0664*E* + 01	**7.4398E + 01**	8.8628*E* + 00	+
F18	1.3888*E* + 04	5.2100*E* + 03	**8.0349E + 03**	2.2066*E* + 03	+
F19	3.6625*E* + 01	3.4612*E* + 00	**3.6208E + 01**	3.0908*E* + 00	+
F20	1.9409*E* + 02	1.4131*E* + 02	**3.4660E + 01**	6.7089*E* + 00	+
F21	3.7093*E* + 02	9.2962*E* + 00	**3.6776E + 02**	1.0301*E* + 01	+
F22	1.0000*E* + 02	1.5498*E* − 13	1.0000*E* + 02	0.0000*E* + 00	=
F23	5.2446*E* + 02	9.2278*E* + 00	**5.1863E + 02**	8.4044*E* + 00	+
F24	5.8819*E* + 02	1.2276*E* + 01	**5.8615E + 02**	8.0042*E* + 00	+
F25	3.8675*E* + 02	2.5661*E* − 02	3.8674*E* + 02	1.1873*E* − 02	=
F26	2.6453*E* + 03	9.4563*E* + 01	**2.5338E + 03**	1.1013*E* + 02	+
F27	**4.9203E + 02**	1.0543*E* + 01	4.9375*E* + 02	8.0730*E* + 00	−
F28	3.2756*E* + 02	4.6815*E* + 01	**3.1464E + 02**	3.5907*E* + 01	+
F29	9.0769*E* + 02	9.6800*E* + 01	**7.3123E + 02**	7.3738*E* + 01	+
F30	1.5880*E* + 04	3.3864*E* + 03	**1.0375E + 04**	1.4700*E* + 03	+
Total numb*E*r of (+/=/−): 24/4/2					

**Table 3:** Comparison results of DE/best/1 on CEC2017 test suite (*D*=30).

	DE/best/1		ESM-DE/best/1		
	Mean	Std.Dev	Mean	Std.Dev	
F1	0.0000*E* + 00	0.0000*E* + 00	0.0000*E* + 00	0.0000*E* + 00	=
F2	2.8258*E* + 10	1.1400*E* + 11	**3.5454E + 04**	1.6884*E* + 05	+
F3	6.0493*E* + 02	3.5493*E* + 02	**3.1110E + 00**	4.8214*E* + 00	+
F4	9.1949*E* + 01	2.7991*E* + 01	9.2586*E* + 01	2.5015*E* + 01	=
F5	9.2763*E* + 01	5.1334*E* + 01	**4.4342E + 01**	1.6997*E* + 01	+
F6	2.5865*E* − 02	6.1852*E* − 02	3.6252*E* − 02	1.1222*E* − 01	=
F7	1.7540*E* + 02	3.2845*E* + 01	**8.2894E + 01**	2.9691*E* + 01	+
F8	9.1332*E* + 01	5.5536*E* + 01	**4.5522E + 01**	1.1158*E* + 01	+
F9	1.3824*E* + 01	2.8355*E* + 01	1.3216*E* + 01	2.0467*E* + 01	=
F10	6.1379*E* + 03	5.6240*E* + 02	**2.2535E + 03**	1.6207*E* + 03	+
F11	6.9152*E* + 01	4.1818*E* + 01	**5.9835E + 01**	3.3934*E* + 01	+
F12	2.4650*E* + 04	1.7178*E* + 04	**1.8722E + 04**	1.1910*E* + 04	+
F13	**8.0829E + 03**	1.3281*E* + 04	1.1159*E* + 04	1.4904*E* + 04	−
F14	1.0157*E* + 02	2.4475*E* + 01	**7.9954E + 01**	3.5773*E* + 01	+
F15	1.3412*E* + 02	7.9017*E* + 01	**8.4741E + 01**	5.4255*E* + 01	+
F16	**4.6549E + 02**	2.8862*E* + 02	4.8784*E* + 02	2.7012*E* + 02	−
F17	1.6363*E* + 02	9.3907*E* + 01	**1.5320E + 02**	8.2527*E* + 01	+
F18	7.5687*E* + 04	5.7404*E* + 04	**4.6144E + 04**	2.9812*E* + 04	+
F19	6.4986*E* + 01	4.8051*E* + 01	**6.0751E + 01**	5.8037*E* + 01	+
F20	1.6043*E* + 02	9.2239*E* + 01	1.6611*E* + 02	9.9222*E* + 01	=
F21	2.8238*E* + 02	4.5823*E* + 01	**2.4758E + 02**	1.1434*E* + 01	+
F22	2.8353*E* + 03	2.8108*E* + 03	**1.2129E + 03**	1.6013*E* + 03	+
F23	4.1270*E* + 02	2.4531*E* + 01	**3.9811E + 02**	1.4860*E* + 01	+
F24	**4.7466E + 02**	2.8842*E* + 01	4.7812*E* + 02	1.3574*E* + 01	−
F25	3.9123*E* + 02	1.1628*E* + 01	3.9129*E* + 02	1.1172*E* + 01	=
F26	1.6222*E* + 03	2.1578*E* + 02	**1.4146E + 03**	4.2851*E* + 02	+
F27	**5.1391E + 02**	1.5952*E* + 01	5.1741*E* + 02	1.2805*E* + 01	−
F28	4.3979*E* + 02	2.9759*E* + 01	**4.2865E + 02**	2.3995*E* + 01	+
F29	**5.7692E + 02**	1.1599*E* + 02	5.9473*E* + 02	1.0825*E* + 02	−
F30	6.3732*E* + 03	3.1823*E* + 03	**4.2434E + 03**	2.5536*E* + 03	+
Total number of (+/=/−): 19/6/5					

**Table 4:** Comparison results of DE/current − to − best/1 on CEC2017 test suite(*D*=30).

	DE/current − to − best/1		ESM-DE/current − to − best/1		
	Mean	Std.Dev	Mean	Std.Dev	
F1	4.2529*E* + 03	1.3861*E* + 04	**2.4194E + 03**	4.8764*E* + 03	+
F2	**1.4541E + 13**	5.2565*E* + 13	7.0969*E* + 13	3.8743*E* + 14	−
F3	2.5651*E* + 02	1.7770*E* + 02	**1.4208E + 00**	2.2041*E* + 00	+
F4	1.1469*E* + 02	1.3856*E* + 01	**1.0468E + 02**	1.8355*E* + 01	+
F5	1.4448*E* + 02	1.2098*E* + 01	**2.9207E + 01**	6.2955*E* + 00	+
F6	7.9340*E* − 03	4.4121*E* − 02	**2.0523E − 03**	1.1284*E* − 02	+
F7	1.7631*E* + 02	1.2016*E* + 01	**7.5749E + 01**	3.9251*E* + 01	+
F8	1.4187*E* + 02	1.2879*E* + 01	**2.7608E + 01**	7.8113*E* + 00	+
F9	4.2194*E* − 01	4.5290*E* − 01	**2.8916E − 01**	3.5412*E* − 01	+
F10	6.1656*E* + 03	3.9958*E* + 02	**4.6688E + 03**	1.8249*E* + 03	+
F11	6.8524*E* + 01	3.1083*E* + 01	**5.5608E + 01**	2.9379*E* + 01	+
F12	2.2269*E* + 04	1.3508*E* + 04	**1.9785E + 04**	1.3868*E* + 04	+
F13	6.5569*E* + 03	3.5373*E* + 03	**4.7370E + 03**	7.3061*E* + 03	+
F14	1.2361*E* + 02	2.9917*E* + 01	**8.7387E + 01**	2.5164*E* + 01	+
F15	2.1775*E* + 02	8.1693*E* + 01	**1.9212E + 02**	8.3283*E* + 01	+
F16	7.4089*E* + 02	2.3808*E* + 02	**4.1651E + 02**	2.2185*E* + 02	+
F17	1.8434*E* + 02	5.3907*E* + 01	**9.5323E + 01**	5.6810*E* + 01	+
F18	5.8340*E* + 04	2.6259*E* + 04	**5.2731E + 04**	3.7186*E* + 04	+
F19	**7.9602E + 01**	2.9727*E* + 01	1.1554*E* + 02	5.1436*E* + 01	−
F20	2.2812*E* + 02	8.2020*E* + 01	**1.2592E + 02**	8.0240*E* + 01	+
F21	3.3232*E* + 02	1.0378*E* + 01	**2.3302E + 02**	1.8782*E* + 01	+
F22	2.9659*E* + 02	1.0908*E* + 03	**1.0053E + 02**	1.1111*E* + 00	+
F23	4.6069*E* + 02	2.1824*E* + 01	**3.8629E + 02**	1.4205*E* + 01	+
F24	5.4998*E* + 02	1.6167*E* + 01	**4.6028E + 02**	1.5954*E* + 01	+
F25	4.0471*E* + 02	1.7220*E* + 01	4.0175*E* + 02	1.7238*E* + 01	=
F26	1.9292*E* + 03	5.1027*E* + 02	**1.3090E + 03**	2.7680*E* + 02	+
F27	5.1686*E* + 02	1.1760*E* + 01	5.1961*E* + 02	1.4919*E* + 01	=
F28	4.7064*E* + 02	3.4376*E* + 01	**4.5744E + 02**	3.0111*E* + 01	+
F29	7.0816*E* + 02	1.0684*E* + 02	**5.2205E + 02**	8.3692*E* + 01	+
F30	9.8358*E* + 03	7.4280*E* + 03	**7.5623E + 03**	3.6244*E* + 03	+
Total number of (+/=/−): 26/2/2					

**Table 5:** Comparison results of j2020 on CEC2017 test suite (*D*=30).

	j2020		ESM-j2020		
	Mean	Std.Dev	Mean	Std.Dev	
F1	**3.9138E − 02**	2.7831*E* − 02	4.1149*E* − 02	2.6801*E* − 02	−
F2	2.1525*E* + 22	7.2306*E* + 22	**3.5391E + 16**	7.4152*E* + 16	+
F3	3.0526*E* + 04	5.6182*E* + 03	**1.5964E + 04**	3.6765*E* + 03	+
F4	**7.6190E + 01**	1.0125*E* + 01	7.7537*E* + 01	1.0041*E* + 01	−
F5	1.5059*E* + 02	1.5882*E* + 01	**3.3617E + 01**	1.1325*E* + 01	+
F6	0.0000*E* + 00	0.0000*E* + 00	0.0000*E* + 00	0.0000*E* + 00	=
F7	1.9215*E* + 02	1.0204*E* + 01	**8.8048E + 01**	2.4532*E* + 01	+
F8	1.5771*E* + 02	9.7337*E* + 00	**3.5822E + 01**	1.3602*E* + 01	+
F9	0.0000*E* + 00	0.0000*E* + 00	0.0000*E* + 00	0.0000*E* + 00	=
F10	6.0724*E* + 03	2.8112*E* + 02	**2.6253E + 03**	9.8373*E* + 02	+
F11	7.1620*E* + 01	2.5123*E* + 01	**1.4904E + 01**	1.6548*E* + 01	+
F12	7.3213*E* + 05	6.1498*E* + 05	**4.9652E + 04**	3.2587*E* + 04	+
F13	3.9600*E* + 02	8.0250*E* + 01	**2.6856E + 02**	4.2577*E* + 01	+
F14	7.1081*E* + 01	5.4527*E* + 00	**5.2870E + 01**	1.1383*E* + 01	+
F15	6.0349*E* + 01	5.8258*E* + 00	**3.6537E + 01**	1.3383*E* + 01	+
F16	9.0601*E* + 02	1.4229*E* + 02	**4.1188E + 02**	1.7961*E* + 02	+
F17	9.1833*E* + 01	3.4540*E* + 01	**4.3249E + 01**	2.4777*E* + 01	+
F18	3.2864*E* + 03	1.5646*E* + 03	**1.5206E + 03**	8.9776*E* + 02	+
F19	3.3522*E* + 01	4.8067*E* + 00	**1.5961E + 01**	2.9754*E* + 00	+
F20	5.3644*E* + 01	2.1292*E* + 01	**4.7332E + 01**	6.8896*E* + 01	+
F21	3.5434*E* + 02	6.7332*E* + 00	**2.3149E + 02**	1.2889*E* + 01	+
F22	1.0000*E* + 02	2.2819*E* − 13	1.0000*E* + 02	8.1676*E* − 14	=
F23	4.9590*E* + 02	6.7701*E* + 00	**3.7594E + 02**	1.0453*E* + 01	+
F24	5.7509*E* + 02	8.0847*E* + 00	**4.5326E + 02**	1.1284*E* + 01	+
F25	3.8677*E* + 02	2.5429*E* − 02	3.8676*E* + 02	1.3848*E* − 02	=
F26	2.3688*E* + 03	1.1160*E* + 02	**1.1769E + 03**	1.2856*E* + 02	+
F27	**4.9473E + 02**	1.1847*E* + 01	4.9543*E* + 02	6.3871*E* + 00	−
F28	3.3192*E* + 02	4.5936*E* + 01	**3.2368E + 02**	4.8265*E* + 01	+
F29	7.8452*E* + 02	8.7484*E* + 01	**4.8871E + 02**	2.6830*E* + 01	+
F30	9.8924*E* + 03	2.2550*E* + 03	**6.4595E + 03**	1.2161*E* + 03	+
Total numb*E*r of (+/=/−): 23/4/3					

**Table 6:** Comparison results of EJADE on CEC2017 test suite (*D*=30).

	EJADE		ESM-EJADE		
	Mean	Std.Dev	Mean	Std.Dev	
F1	0.0000*E* + 00	0.0000*E* + 00	0.0000*E* + 00	0.0000*E* + 00	=
F2	**4.5316E + 03**	2.1794*E* + 04	1.2673*E* + 04	5.6411*E* + 04	−
F3	0.0000*E* + 00	0.0000*E* + 00	0.0000*E* + 00	0.0000*E* + 00	=
F4	**2.4107E + 01**	2.9037*E* + 01	2.6740*E* + 01	2.9430*E* + 01	−
F5	1.8962*E* + 01	4.4630*E* + 00	**1.7560E + 01**	4.1722*E* + 00	+
F6	**6.9845E − 07**	2.4275*E* − 06	1.4467*E* − 06	6.4530*E* − 06	−
F7	4.6647*E* + 01	2.8902*E* + 00	**4.5802E + 01**	3.2092*E* + 00	+
F8	1.8850*E* + 01	3.1904*E* + 00	**1.7218E + 01**	5.1087*E* + 00	+
F9	1.9587*E* − 01	3.4505*E* − 01	**1.2548E − 01**	1.8845*E* − 01	+
F10	1.8437*E* + 03	3.0553*E* + 02	**1.5558E + 03**	5.3726*E* + 02	+
F11	4.7182*E* + 01	2.6517*E* + 01	**3.9138E + 01**	2.3463*E* + 01	+
F12	2.0542*E* + 03	1.8865*E* + 03	**1.8759E + 03**	1.2938*E* + 03	+
F13	4.9648*E* + 01	3.1191*E* + 01	**3.7663E + 01**	1.7336*E* + 01	+
F14	3.9862*E* + 01	1.3835*E* + 01	**3.8508E + 01**	1.1120*E* + 01	+
F15	**4.1678E + 01**	4.0043*E* + 01	4.2540*E* + 01	3.6838*E* + 01	−
F16	3.2052*E* + 02	1.8067*E* + 02	**2.6554E + 02**	1.5914*E* + 02	+
F17	5.7500*E* + 01	1.3465*E* + 01	**5.1900E + 01**	3.5779*E* + 01	+
F18	**7.7806*E* + 01**	5.1375*E* + 01	8.8347*E* + 01	6.3700*E* + 01	−
F19	**1.7808E + 01**	1.0382*E* + 01	1.9606*E* + 01	1.8146 *E* + 01	−
F20	1.0632*E* + 02	5.7664*E* + 01	**5.1910E + 01**	5.3314*E* + 01	+
F21	2.1850*E* + 02	3.6330*E* + 00	**2.1748E + 02**	4.0920*E* + 00	+
F22	**1.0008E + 02**	4.4179*E* − 01	1.6606*E* + 02	3.6781*E* + 02	−
F23	3.6253*E* + 02	6.0195*E* + 00	**3.6162E + 02**	6.1887*E* + 00	+
F24	4.3448*E* + 02	6.1456*E* + 00	4.3460*E* + 02	5.0372*E* + 00	=
F25	3.8703*E* + 02	7.8427*E* − 01	3.8701*E* + 02	3.6023*E* − 01	=
F26	1.1198*E* + 03	7.2825*E* + 01	**1.0310E + 03**	2.7881*E* + 02	+
F27	5.0690*E* + 02	8.8147*E* + 00	5.0681*E* + 02	6.6749*E* + 00	=
F28	3.7620*E* + 02	6.0263*E* + 01	**3.3258E + 02**	5.2414*E* + 01	+
F29	4.6359*E* + 02	2.7079*E* + 01	**4.4343E + 02**	3.0038*E* + 01	+
F30	2.1586*E* + 03	1.9675*E* + 02	**2.1400E + 03**	2.0292*E* + 02	+
Total number of (+/ = /−): 18/5/7					

**Table 7:** Comparison results of BeSD on CEC2017 test suite (*D*=30).

	BeSD		ESM- BeSD		
	Mean	Std.Dev	Mean	Std.Dev	
F1	0.0000*E* + 00	0.0000*E* + 00	0.0000*E* + 00	0.0000*E* + 00	=
F2	7.2258*E* + 00	1.9085*E* + 01	**3.9032E + 00**	1.4996*E* + 01	+
F3	0.0000*E* + 00	0.0000*E* + 00	0.0000*E* + 00	0.0000*E* + 00	=
F4	**6.8185E + 00**	1.6230*E* + 01	1.7131*E* + 01	2.6599*E* + 01	−
F5	4.5762*E* + 01	2.8543*E* + 01	**2.7217E + 01**	8.1867*E* + 00	+
F6	0.0000*E* + 00	0.0000*E* + 00	1.7219*E* − 07	3.7418*E* − 07	=
F7	1.3363*E* + 02	1.5180*E* + 01	**5.1262E + 01**	6.7117*E* + 00	+
F8	5.6343*E* + 01	2.9912*E* + 01	**2.6864E + 01**	7.8132*E* + 00	+
F9	6.1295*E* − 02	1.9377*E* − 01	6.7718*E* − 02	1.7464*E* − 01	=
F10	5.8972*E* + 03	3.7315*E* + 02	**2.5464E + 03**	5.6311*E* + 02	+
F11	**2.6612E + 01**	1.5396*E* + 01	3.7557*E* + 01	2.2784*E* + 01	−
F12	**8.3133E + 03**	4.3141*E* + 03	8.9538*E* + 03	7.0576*E* + 03	−
F13	2.2656*E* + 02	2.3629*E* + 02	**6.3469E + 01**	2.8828*E* + 01	+
F14	6.1777*E* + 01	1.5857*E* + 01	**4.1127E + 01**	9.6938*E* + 00	+
F15	6.2060*E* + 01	3.5382*E* + 01	**3.8429E + 01**	2.6456*E* + 01	+
F16	6.1366*E* + 02	1.7387*E* + 02	**2.2941E + 02**	1.8203*E* + 02	+
F17	1.0414*E* + 02	2.0838*E* + 01	**3.4358E + 01**	1.1387*E* + 01	+
F18	2.1008*E* + 02	9.9628*E* + 01	**7.7754E + 01**	4.7271*E* + 01	+
F19	4.0368*E* + 01	2.1662*E* + 01	**3.0129E + 01**	1.4941*E* + 01	+
F20	1.1522*E* + 02	4.3894*E* + 01	**4.3747E + 01**	5.1852*E* + 01	+
F21	2.3501*E* + 02	2.5203*E* + 01	**2.2421E + 02**	9.6408*E* + 00	+
F22	1.0000*E* + 02	1.1357*E* − 13	1.0000*E* + 02	2.2124*E* − 13	=
F23	3.7384*E* + 02	1.6564*E* + 01	3.7369*E* + 02	9.3329*E* + 00	=
F24	4.4032*E* + 02	7.1928*E* + 00	**4.3973E + 02**	8.2282*E* + 00	+
F25	3.8708*E* + 02	2.9529*E* − 01	3.8709*E* + 02	2.7823*E* − 01	=
F26	1.1447*E* + 03	1.7473*E* + 02	**1.0790E + 03**	2.7897*E* + 02	+
F27	**5.0192E + 02**	5.4148*E* + 00	5.0552*E* + 02	8.0174*E* + 00	−
F28	**3.1676E + 02**	3.8969*E* + 01	3.3033*E* + 02	4.8241*E* + 01	−
F29	5.9423*E* + 02	7.0224*E* + 01	**4.4240E + 02**	3.4337*E* + 01	+
F30	2.5624*E* + 03	3.5089*E* + 02	**2.1699E + 03**	1.3768*E* + 02	+
Total number of (+/ = /−): 18/7/5					

**Table 8:** Comparison results of EBSHADE on CEC2017 test suite (*D*=30).

	EBSHADE		ESM-EBSHADE		
	Mean	Std.Dev	Mean	Std.Dev	
F1	0.0000*E* + 00	0.0000*E* + 00	0.0000*E* + 00	0.0000*E* + 00	=
F2	1.9972*E* + 04	1.1037*E* + 05	**1.6852E + 02**	7.3798*E* + 02	+
F3	0.0000*E* + 00	0.0000*E* + 00	0.0000*E* + 00	0.0000*E* + 00	=
F4	5.6852*E* + 01	1.0598*E* + 01	**5.3253E + 01**	1.7773*E* + 01	+
F5	**1.5508E + 01**	2.5802*E* + 00	1.6273*E* + 01	6.3554*E* + 00	−
F6	**6.8453E − 08**	1.0785*E*-07	4.2769*E *** − **07	7.4531*E *** − **07	−
F7	4.6604*E* + 01	2.7607*E* + 00	**4.6489E + 01**	4.8631*E* + 00	+
F8	1.6879*E* + 01	2.8304*E* + 00	**1.6186E + 01**	3.4825*E* + 00	+
F9	1.7544*E* − 02	8.2641*E *** − **02	**1.4656E − 02**	8.1599*E *** − **02	+
F10	1.6353*E* + 03	2.3269*E* + 02	**1.4154E + 03**	2.6504*E* + 02	+
F11	2.7724*E* + 01	2.6071*E* + 01	**2.6265E + 01**	2.5887*E* + 01	+
F12	1.2219*E* + 03	4.6754*E* + 02	**1.2126E + 03**	4.4003*E* + 02	+
F13	3.8215*E* + 01	2.0185*E* + 01	**3.7691E + 01**	1.4370*E* + 01	+
F14	**2.8375E + 01**	8.7055*E* + 00	3.0017*E* + 01	4.8689*E* + 00	−
F15	**1.7726E + 01**	1.0888*E* + 01	2.1258*E* + 01	1.2917*E* + 01	−
F16	2.9276*E* + 02	1.1820*E* + 02	**2.0707E + 02**	1.2958*E* + 02	+
F17	4.5658*E* + 01	1.0476*E* + 01	3.5719*E* + 01	1.0310*E* + 01	=
F18	**9.0145E + 01**	4.7859*E* + 01	9.4472*E* + 01	5.6305*E* + 01	−
F19	1.4666*E* + 01	8.9947*E* + 00	**1.2179E + 01**	4.1749*E* + 00	+
F20	6.3748*E* + 01	4.4406*E* + 01	**5.2312E + 01**	4.9270*E* + 01	+
F21	**2.1777E + 02**	3.4870*E* + 00	2.1847*E* + 02	4.1234*E* + 00	−
F22	1.0051*E* + 02	2.8400*E* + 00	1.0000*E* + 02	2.2124*E *** − **13	=
F23	3.6665*E* + 02	5.5240*E* + 00	**3.6453E + 02**	5.2131*E* + 00	+
F24	4.3676*E* + 02	3.7374*E* + 00	4.3642*E* + 02	6.0049*E* + 00	=
F25	3.8681*E* + 02	5.8187*E*-02	3.8684*E* + 02	7.5494*E *** − **02	=
F26	1.1214*E* + 03	6.7789*E* + 01	**1.1160E + 03**	7.3256*E* + 01	+
F27	5.0632*E* + 02	4.6020*E* + 00	**5.0579E + 02**	6.3243*E* + 00	+
F28	3.4497*E* + 02	5.8589*E* + 01	**3.4205E + 02**	5.8955*E* + 01	+
F29	4.6900*E* + 02	1.3609*E* + 01	**4.4219E + 02**	2.2017*E* + 01	+
F30	2.0864*E* + 03	1.3828*E* + 02	**2.0213E + 03**	1.6473*E* + 02	+
Total number of (+/ = /−): 18/6/6					

**Table 9:** Comparison results of EBLSHADE on CEC2017 test suite (*D*=30).

	EBLSHADE		ESM-EBLSHADE		
	Mean	Std.Dev	Mean	Std.Dev	
F1	0.0000*E* + 00	0.0000*E* + 00	0.0000*E* + 00	0.0000*E* + 00	=
F2	0.0000*E* + 00	0.0000*E* + 00	0.0000*E* + 00	0.0000*E* + 00	=
F3	0.0000*E* + 00	0.0000*E* + 00	0.0000*E* + 00	0.0000*E* + 00	=
F4	5.8562*E* + 01	3.4396*E* − 14	5.8562*E* + 01	4.0776*E* − 14	=
F5	6.6978*E* + 00	1.3325*E* + 00	6.9719*E* + 00	1.5418*E* + 00	=
F6	0.0000*E* + 00	0.0000*E* + 00	1.4398*E* − 08	4.1347*E* − 08	=
F7	3.7938*E* + 01	1.3728*E* + 00	**3.6957E + 01**	1.2423*E* + 00	+
F8	7.4627*E* + 00	1.5388*E* + 00	**6.9654E + 00**	1.6395*E* + 00	+
F9	0.0000*E* + 00	0.0000*E* + 00	0.0000*E* + 00	0.0000*E* + 00	=
F10	1.3903*E* + 03	2.5407*E* + 02	**1.2476E + 03**	2.3487*E* + 02	+
F11	**3.3748E + 01**	2.8992*E* + 01	4.0898*E* + 01	2.8428*E* + 01	−
F12	1.1107*E* + 03	3.2096*E* + 02	**1.1021E + 03**	3.2418*E* + 02	+
F13	1.8932*E* + 01	7.7131*E* + 00	**1.6355E + 01**	5.4803*E* + 00	+
F14	2.1636*E* + 01	1.1516*E* + 00	**2.1493E + 01**	3.6187*E* + 00	+
F15	3.9785*E* + 00	1.8483*E* + 00	**3.6417E + 00**	1.6998*E* + 00	+
F16	4.1583*E* + 01	3.4857*E* + 01	**2.0657E + 01**	5.3775*E* + 00	+
F17	3.1880*E* + 01	5.6772*E* + 00	**3.1060E + 01**	4.8829*E* + 00	+
F18	**2.2262E + 01**	1.5977*E* + 00	2.2496*E* + 01	1.2879*E* + 00	−
F19	6.2724*E* + 00	2.2466*E* + 00	**5.6099E + 00**	1.3474*E* + 00	+
F20	3.2672*E* + 01	6.5190*E* + 00	**2.7681E + 01**	6.2855*E* + 00	+
F21	2.0835*E* + 02	1.4350*E* + 00	**2.0819E + 02**	1.4361*E* + 00	+
F22	1.0000*E* + 02	0.0000*E* + 00	1.0000*E* + 02	8.1676*E* − 14	=
F23	**3.5311E + 02**	3.0584*E* + 00	3.5407*E* + 02	2.9996*E* + 00	−
F24	**4.2796E + 02**	1.7733*E* + 00	4.2807*E* + 02	1.8546*E* + 00	−
F25	3.8672*E* + 02	1.9899*E* − 02	3.8672*E* + 02	2.4427*E* − 02	=
F26	9.7950*E* + 02	4.0916*E* + 01	**9.7385E + 02**	2.9174*E* + 01	+
F27	5.0689*E* + 02	5.5012*E* + 00	**5.0580E + 02**	4.6504*E* + 00	+
F28	3.5816*E* + 02	6.2765*E* + 01	**3.2838E + 02**	4.8986*E* + 01	+
F29	4.3621*E* + 02	7.9105*E* + 00	**4.3392E + 02**	6.5290*E* + 00	+
F30	**1.9967E + 03**	7.3147*E* + 01	2.0385*E* + 03	8.6201*E* + 01	−
Total number of (+/ = /−): 16/9/5					

**Table 10:** Results obtained by the Wilcoxon test.

Algorithms	*R*+	*R*−	*P*-value
ESM-DE/rand/1 vs. DE/rand/1	**446.5**	18.5	**0.039**
ESM-DE/best/1 vs. DE/best/1	**376.5**	88.5	**0.047**
ESM-DE/current − to − best/1 vs. DE/current − to − best/1	**423**	42	**0.031**
ESM-j2020 vs. j2020	**419.5**	45.5	**0.038**
ESM-EJADE vs. EJADE	**326.5**	138.5	0.081
ESM-BeSD vs. BeSD	**338.5**	126.5	**0.046**
ESM-EBSHADE vs. EBSHADE	**375.5**	89.5	0.075
ESM-EBLSHADE vs. EBLSHADE	**341.5**	123.5	0.089

**Table 11:** The classification statistics result of proposed ESM in eight algorithms.

Functions	+(%)	=(%)	−(%)
Unimodal functions	45.83	41.67	12.50
Simple multimodal functions	64.29	23.21	12.50
Hybrid functions	**81.25**	2.50	16.25
Composition functions	62.50	22.50	15.00

**Table 12:** The B-W values of all ESM-based algorithms on CEC2017 under different *Q* values.

Algorithms	*Q*=100	*Q*=110	*Q*=120	*Q*=130	*Q*=140
ESM-DE/rand/1	22	21	22	23	20
ESM-DE/best/1	13	14	14	13	14
ESM-DE/current − to − best/1	24	24	24	22	20
ESM-j2020	18	21	20	19	17
ESM-EJADE	10	12	11	10	10
ESM-BeSD	11	10	13	12	12
ESM-EBSHADE	10	11	12	10	11
ESM-EBLSHADE	9	10	11	10	10
Total	117	126	**127**	119	114

**Table 13:** The statistical results of all ESM-based algorithms on CEC2017 under different dimensions.

Algorithms	*D*=10	*D*=30	*D*=50	*D*=100
ESM-DE/rand/1 vs. DE/rand/1	23/5/2	24/4/2	22/6/2	25/4/1
ESM-DE/best/1 vs. DE/best/1	17/8/5	19/6/5	18/6/6	19/5/6
ESM-DE/current − to − best/1 vs. DE/current − to − best/1	23/4/3	26/2/2	25/2/3	23/5/2
ESM-j2020 vs. j2020	22/5/3	23/4/3	21/5/4	22/4/4
ESM-EJADE vs. EJADE	16/8/6	18/5/7	20/5/5	19/5/6
ESM-BeSD vs. BeSD	17/7/6	18/7/5	19/6/5	18/5/7
ESM-EBSHADE vs. EBSHADE	16/9/5	18/6/6	18/5/7	17/6/7
ESM-EBLSHADE vs. EBLSHADE	15/10/5	16/9/5	15/11/4	16/8/6

**Table 14:** The Friedman test results of all algorithms on CEC2017 under different dimensions.

Algorithms	*D*=10	*D*=30	*D*=50	*D*=100	Mean ranking	Rank
ESM-EBLSHADE	**2.13**	**2.62**	**2.88**	**2.57**	**10.20**	**1**
EBLSHADE	2.98	3.03	3.21	3.55	12.77	2
ESM-EBSHADE	4.85	4.78	5.01	4.89	19.53	3
EBSHADE	4.98	5.73	5.55	5.21	21.47	4
ESM-EJADE	5.89	6.07	6.24	5.87	24.07	5
ESM-BeSD	5.31	6.73	6.84	6.57	25.45	6
EJADE	6.51	6.93	6.95	6.88	27.27	7
ESM-j2020	7.51	7.62	7.58	7.41	30.12	8
BeSD	7.11	7.93	8.02	8.57	31.63	9
j2020	9.82	10.89	9.58	11.21	41.50	10
ESM-DE/rand/1	10.09	11.03	11.59	11.07	43.78	11
ESM-DE/current − to − best/1	10.55	11.63	11.21	11.85	45.24	12
ESM-DE/best/1	10.11	11.82	11.85	11.98	45.76	13
DE/best/1	11.53	12.65	12.33	12.57	49.08	14
DE/rand/1	12.31	12.70	12.58	12.34	49.93	15
DE/current − to − best/1	11.89	13.83	12.89	12.59	51.20	16
Friedman-P-value	0.00	0.00	0.00	0.00		

## Data Availability

The data that support the findings of this study are available from the corresponding author upon reasonable request.
